# A Rare Etiology of Unilateral Pseudo-Pulmonary Fibrosis in a Puerto Rican Child

**DOI:** 10.7759/cureus.16473

**Published:** 2021-07-19

**Authors:** Daniela M Albors-Agulló, Pedro Diaz Ortiz, Wilfredo De Jesús-Rojas

**Affiliations:** 1 Pediatrics, Ponce Health Sciences Universtiy, Ponce, PRI; 2 Radiology, Ponce Health Sciences University, Ponce, PRI; 3 Pediatrics, Ponce Health Science University School of Medicine, Ponce, PRI

**Keywords:** pseudo-pulmonary fibrosis, congenital pulmonary artery hypoplasia, 3-methylglutagonic aciduria, recurrent respiratory tract infections, chronic wet cough

## Abstract

Congenital unilateral pulmonary hypoplasia of a pulmonary artery is considered a rare congenital anomaly in the pediatric and adult population. With an estimated prevalence of one in 200,000, it can range from partial to near-total lung underdevelopment. The diagnosis of lung and pulmonary artery hypoplasia is challenging in adults as they can easily be mistaken for more common diseases. Many survive into adulthood with minimal or no symptoms, which makes their identification challenging. We present the case of a 14-year-old female with a previous diagnosis of 3-methylglutaconic aciduria (3-MGA-uria) with a history of chronic wet cough andrecurrent respiratory tract infections (RTIs) that led to multiple hospitalizations throughout her childhood. After further evaluation, the patient was diagnosed with hypoplasia of the right-sided pulmonary artery system and its branches. This case report highlights the importance of early identification of congenital unilateral pulmonary hypoplasia of a pulmonary artery to prevent pulmonary complications like recurrent RTIs in pediatric patients with rare diseases.

## Introduction

Congenital unilateral pulmonary hypoplasia of a pulmonary artery is rare congenital anomalies [1]. With an estimated prevalence of one in 200,000 [1-3], pulmonary hypoplasia can range from partial to near-total lung underdevelopment. In 1995, Boyden classified pulmonary hypoplasia as a variable amount of lung parenchyma, bronchial tree, and supporting vasculature [4]. Due to embryologic relationships, pulmonary artery agenesis commonly occurs on the side of the chest opposite to the aortic arch [5]. The distal intrapulmonary branches of the affected artery usually remain intact and receive collateral supply from bronchial, intercostal, internal mammary, subdiaphragmatic, subclavian, and even the coronary arteries [6]. As a result of diminished blood supply, the lung on the affected side is usually small and hypoplastic [6]. An appearance termed “pseudofibrosis” is sometimes seen in the affected lung apex due to the formation of transpleural collateral vessels between peripheral pulmonary arterial branches and systemic arteries [7].

Reported symptoms include dyspnea, cough, recurrent hemoptysis, recurrent respiratory tract infections (RTIs), tachycardia, and pulmonary hypertension (PHT) [1-6]. A substantial portion of congenital lung anomalies is detected early in childhood, frequently prenatally. Diagnosis is usually made in the setting of severe respiratory insufficiency or with the occurrence of acute respiratory infections later in childhood [8]. However, lung and pulmonary artery hypoplasia diagnosis is challenging in adults as they can easily be mistaken for more common diseases [4], and many patients survive into adulthood with minimal or no symptoms [9].

This report focuses on a rare etiology of unilateral pulmonary hypoplasia with an associated history of chronic lung disease presenting in a 14-year-old patient with 3-methylglutaconic aciduria (3-MGA-uria). We explore the potential etiologies for the development of unilateral pseudo-pulmonary fibrosis. This article was previously presented as a meeting abstract at the 2021 American Thoracic Society Virtual Conference on May 14-19, 2021.

## Case presentation

A 14-year-old Hispanic female diagnosed with 3-MGA-uria type 1 presented with her caretaker to our institution with complaints of chronic wet cough and recurrent RTIs since she was three years old that have led to multiple hospitalizations throughout her childhood. The patient was born at 40-week gestation, weighing 4lb, 14oz via cesarean section due to failure of labor progression. She was admitted to the Neonatal Intensive Care Unit (NICU) due to hyperbilirubinemia and discharged without complications. At one month old, the patient started having daily seizures, which were diagnosed as infantile spasms. The patient started demonstrating delays in developmental history, dysphagia, dystonia, psychomotor retardation, and failure to thrive during routine examinations. The patient was referred to a nutritionist, occupational, speech, and physical therapist, with minimal improvement in her condition. After a comprehensive genetic and metabolic evaluation, the patient was diagnosed with 3-MGA-uria type 1 at eight months old. Positive findings on her examination included: a small head, sunken eyes, and feet inversion. The patient body mass index (BMI) is 15.7 kg/m^2^ (third BMI percentile) with a poor skeletal build. The respiratory evaluation was pertinent for intermittent episodes of wet cough and asymmetrical breath sounds with intermittent central rhonchi. Mild scoliosis and hypotonia in all extremities were observed.

**Figure 1:**
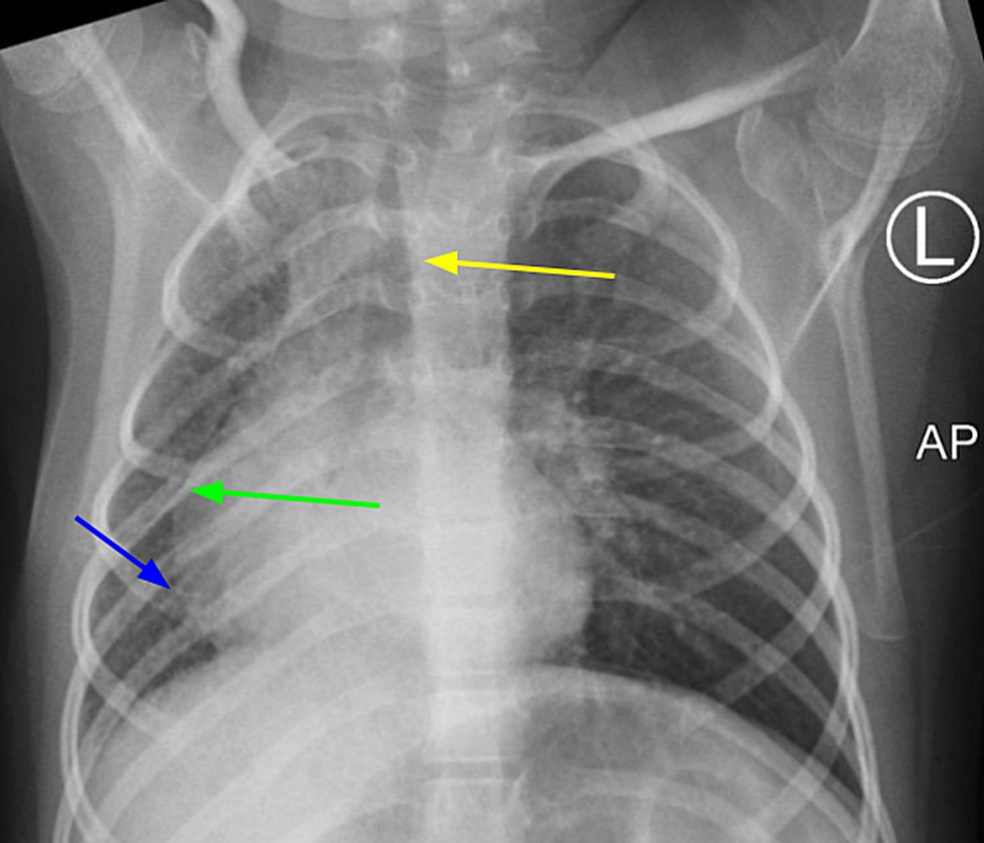
Anteroposterior chest x-ray (CXR) evaluating causes for chronic wet cough Anteroposterior (AP) CXR demonstrated nonspecific thickening of the interstitial markings more confluent at the right pulmonary field (blue arrow). Mediastinum was shifted to the right side (green arrow). The tracheal deviation was noted (yellow arrow).

Chest x-ray (CXR) (Figure 1) demonstrated non-specific thickening of the interstitial markings. Echocardiography demonstrated mild tricuspid regurgitation by congenital defect and was negative for PHT. Diaphragmatic ultrasound (US) showed the adequate movement of both hemidiaphragm.

**Figure 2:**
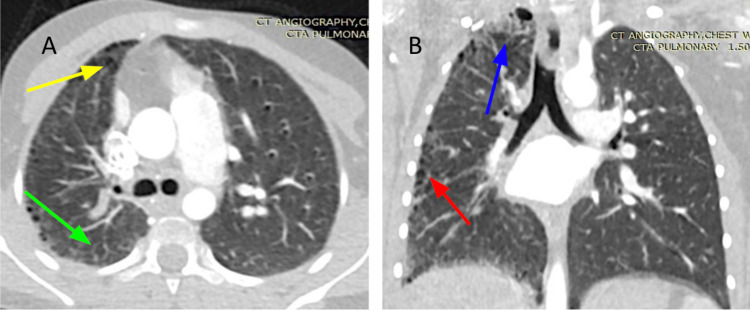
Contrast-enhanced computer tomography (CECT) of the thorax (A) Axial image of a CECT of the midlung zones showing subpleural honeycombing pattern configuration at the right-sided lung parenchyma (yellow arrow). Unilateral right-sided pseudo-fibrosis type changes present (green arrow). (B) Coronal image of a CECT image of the thorax demonstrated multifocal and radiolucent destructive-like pattern configuration on the right pulmonary parenchyma. There were curvilinear areas of mild bronchiectasis changes noted at the right upper lobe (blue arrow). Also, multifocal reticular interstitial opacities and cystic lucencies extending at the periphery of the lung were present (red arrow).

For further evaluation and diagnosis, a contrast-enhanced computer tomography (CECT) of the thorax using multiple axial images (Figures 2A, 2B) was ordered. Right pulmonary parenchyma demonstrated multifocal and radiolucent destructive-like pattern configuration. There were curvilinear areas of mild bronchiectasis changes noted at the right upper lobe. Also, multifocal reticular interstitial opacities and cystic lucencies extending at the periphery of the lung were present. Subpleural honeycombing pattern configurations were present at the right-sided pulmonary parenchyma. Asymmetry of the thorax with a smaller right lung and slight elevation of the right-sided diaphragm were also observed. Mild diffuse bronchial wall thickening changes are noted related to mild bronchial wall inflammatory disease.

**Figure 3:**
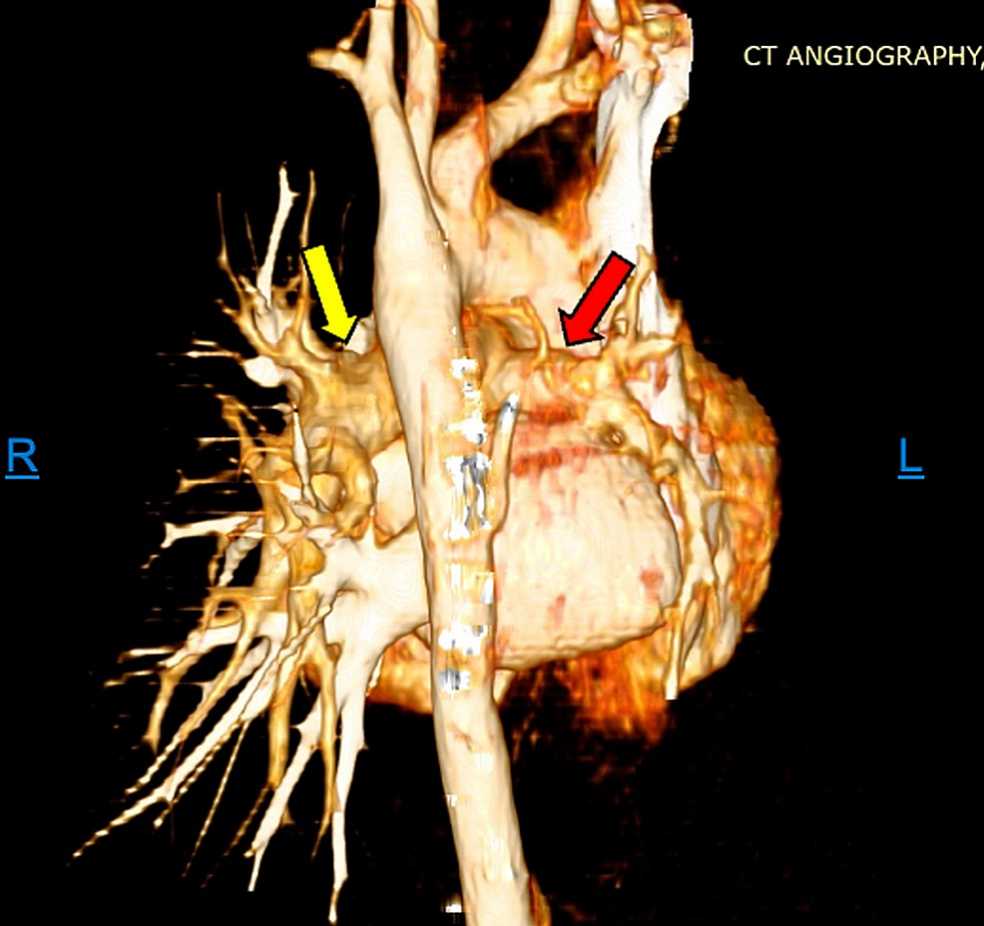
Three-dimensional reconstruction of pulmonary arteries Posterior view of the pulmonary arterial circulation. The main pulmonary truck measured 1.73 cm in maximal transverse diameter. The right main pulmonary artery was noted to be hypoplastic (8 mm) (red arrow) as compared with the left pulmonary artery (1.5 cm) (yellow arrow).

Three-dimensional (3D) reconstruction images of the cardiovascular and coronal reconstruction were performed (Figure 3). The main pulmonary truck measured 1.73 cm in maximal transverse diameter. The right main pulmonary artery was noted to be hypoplastic (8 mm) as compared with the left pulmonary artery (1.5 cm). Right upper and lower lobes arteries were hypoplastic.

All findings on radiologic imaging were consistent with hypoplasia of the right-sided pulmonary artery system and branches with unilateral right-sided pseudo pulmonary fibrosis type changes.

## Discussion

The patient was diagnosed with hypoplasia of the right-sided pulmonary artery system and its branches. Differential diagnosis includes: Swyer-James-MacLeod’s syndrome (SJMS), lobar atelectasis, post-lobectomy status, and compensatory emphysema, and pulmonary thromboembolic disease can have a similar radiographic appearance [6,9]. The symptoms and clinical manifestations associated with hypoplasia of a pulmonary artery may vary between patients. The presence of cough, dyspnea, recurrent RTIs, PHT, hemoptysis, and pulmonary hypoplasia findings are some of them.

Typical findings on a plain chest radiographic image of unilateral pulmonary artery agenesis can be subtle but may include ipsilateral displacement of heart and mediastinum, absent hilar shadow, and volume loss of affected lung with hyperinflation of the contralateral lung [8]. Our patient’s CXR demonstrated volume loss of the affected right lung but no hyperinflation of the contralateral lung nor other additional changes.

When CXR is suspicious, the diagnosis of pulmonary artery hypoplasia can be confirmed by a CECT scan of the thorax, magnetic resonance imaging, or transthoracic echocardiography [6]. This noninvasive test was also used to evaluate the presence and extent of bronchial and pulmonary thickening lesions changes in our patient. CECT imaging revealed the marked hypoplasia of the right-sided pulmonary artery system and branches and areas of pseudo-fibrotic-like changes on the affected lung, which was concerning for pseudo-fibrosis. Echocardiography is necessary to exclude any other cardiac abnormalities and to evaluate the presence of associated PHT, which may preclude long-term survival [3,9]. As aforementioned, echocardiography was negative for PHT in our patient. Pulmonary angiography is the gold standard investigation for the diagnosis of pulmonary artery agenesis and estimation of collateral circulation [6]. However, being an invasive procedure, it is better advised when a patient is planned for arterial embolization [6,7]. 

Lack of arterial blood flow to the affected lung in pulmonary artery hypoplasia can result in poor delivery of inflammatory cells to sites of inflammation and impair ciliary function [5]. In addition, poor blood flow to the affected lung may result in alveolar hypocapnia, leading to secondary bronchoconstriction and mucous trapping increasing host susceptibility to bacterial and viral proliferation [3,5], which may be the cause of the multiple respiratory infections and hospitalization in our patient’s case. Chronic infection can lead to bronchiectasis in some cases [4]. Limited blood flow to the patient’s right lung parenchyma may restrict its development and accentuate pseudo-fibrotic changes seen in our patient’s hypoplastic lung. Thus, pseudo-fibrosis can result from the presence of multifocal reticular interstitial opacities in a hypoplastic lung with a limited blood supply, which alters the parenchymal structure.

An early diagnosis is extremely important because prognosis depends on the presence of many complications such as pulmonary infections, pulmonary hemorrhage, and, especially, PHT [3,4,6,7]. The overall mortality rate in UAPA is 7% [3,6,7]. Common causes of mortality include right heart failure, respiratory failure, massive hemoptysis, and high-altitude pulmonary edema [6,7]. There are no guidelines or consensus regarding treatment [3,6]. For asymptomatic patients, yearly echocardiography evaluation is advised to rule out PHT [4,6,9]. Vasodilator therapy is advisable for patients with PHT [4,6,7]. Revascularization of the peripheral branches of the affected pulmonary artery to the pulmonary hilum has been attempted successfully and has yielded better results in the pediatric population [6]. For patients presenting with massive hemoptysis or with recurrent severe respiratory infections, surgical resection of the affected lung may be needed. Pulmonary artery embolization is an alternate option for patients not fit for surgery [6].

Follow-up with subspecialists should be considered for timely detection and treatment of complications, especially PHT secondary to unilateral pulmonary hypoplasia (Table 1).

**Table 1:** Multidisciplinary approach to manage Unilateral Pulmonary Hypoplasia in Pediatrics Pulmonary Hypertension (PHT), Respiratory Tract Infections (RTIs), American Academy of Pediatrics (AAP), Centers for Disease Control and Prevention (CDC), Chest X-Ray (CXR), Contrast-enhanced Computer Tomography (CECT).

Pediatric Subspecialty	Screening	Evaluation
Cardiology	Exclude concomitant cardiac anomalies and evaluation of the presence of PHT.	Yearly echocardiography evaluation to rule out PHT. Management of PHT (vasodilator therapy is advised if needed).
Genetics	Diagnostic testing for underlining metabolic or congenital heart diseases.	Referral of caretakers to a genetic counselor for diagnostic discussion, prognosis, and family planning.
Primary Care	Detection of chronic cough, recurrent RTIs.	Follow immunization schedule, including Influenza, Pneumococcal polysaccharide vaccine (PPSV23), and SARS-CoV-2 vaccines as per AAP and CDC guidelines.
Pulmonology	Explore etiologies of chronic cough, recurrent RTIs, hemoptysis, and dyspnea. Rule out aspiration into the airway.	Complete pulmonary function tests if able to complete. Suspicion of pseudofibrosis on CXR and CECT. Management and treatment of associated pulmonary symptoms and complications. Work alongside cardiology for the management of PHT.
Radiology	Suspicion of pseudofibrosis on CXR.	Confirmation of diagnosis by CECT scan of thorax, magnetic resonance imaging, or transthoracic echocardiography.

## Conclusions

This case report highlights the difficulty of identifying and diagnosing unilateral pulmonary hypoplasia due to its variable symptomatic presentation in children. Also, it recognizes the importance of early identification of the defect to prevent the worsening of complications such as recurrent pulmonary infections, irreversible bronchiectasis, and pseudo-pulmonary fibrosis in pediatrics. Early recognition of unilateral pulmonary hypoplasia and individualized treatment for associated complications may improve prognosis. Additional studies on how early interventions and treatment may change the natural history of this disease are needed.
